# An Investigation on High-Resolution Temperature Measurement in Precision Fly-Cutting

**DOI:** 10.3390/s21041530

**Published:** 2021-02-23

**Authors:** Daniel Gräbner, Tjarden Zielinski, Andrey Vovk, Oltmann Riemer, Bernhard Karpuschewski, Walter Lang

**Affiliations:** 1 Institute for Microsensors, Actuators and Systems (IMSAS), University of Bremen, 28359 Bremen, Germany; wlang@imsas.uni-bremen.de; 2 Leibniz-Institut für Werkstofforientierte Technologien—IWT, 28359 Bremen, Germany; zielinski@iwt.uni-bremen.de (T.Z.); vovk@iwt-bremen.de (A.V.); riemer@iwt.uni-bremen.de (O.R.); karpuschewski@iwt-bremen.de (B.K.); 3 MAPEX Center for Materials and Processes, University of Bremen, 28359 Bremen, Germany

**Keywords:** temperature measurement, thermocouple, precision machining, fly-cutting, machining simulation, Johnson Cook model

## Abstract

The loads acting on a workpiece during machining processes determine the modification of the surface of the final workpiece and, thus, its functional properties. In this work, a method that uses thermocouples to measure the temperature in precision fly-cutting machining with high spatial and temporal resolution is presented. Experiments were conducted for various materials and machining parameters. We compare experimental measurement data with results from modern and advanced machining process simulation and find a good match between experimental and simulation results. Therefore, the simulation is validated by experimental data and can be used to calculate realistic internal loads of machining processes.

## 1. Introduction

High-precision milling and cutting have been long established in industrial production, and a lot of research has been conducted on these processes as well. The quality of machined parts has been improved over a long period of time regarding quantities such as dimensional accuracy or surface roughness. However, the exact impact of machining processes on the surface properties of the machined parts is often uncertain before machining and subsequently adjusting the process parameters. The state of the surface and subsurface layer after machining is referred to as surface integrity [1]. It is of great interest to understand and describe material modification mechanisms due to machining in order to be able to predict surface properties beforehand. To understand the material modification, the local process load on the machined part has to be determined. The interdisciplinary Transregional Collaborative Research Center (SFB TRR) 136 “Process Signatures” strives to investigate various machining processes and to analyze the process loads and material modification mechanisms that mainly determine the surface integrity of machined parts [2,3,4]. To accommodate for the solution of the inverse problem and prediction of surface properties, the temperature and the local and temporal temperature gradient were identified as a local material load. This investigation aims to validate the simulation and the material model generated in the SFB TRR with a highly local temperature measurement as near to the machining zone as possible. The generated heat of the comparably small process is expected to only generate high temperatures locally, as seen in [5].

Davies et al. provide a detailed overview on various methods for temperature measurement in machining processes [6]. Nowadays, temperature measurement is generally performed by thermographic imaging or by commercially available thermocouples. Thermography is mostly suitable for the measurement of temperature on surfaces [7] but can also be used to measure temperature in subsurface areas [8]. Thermocouples can either be used to monitor the temperature of the workpiece [9] or the tool [10].

Precision cutting is a finishing process, which gives a workpiece its final shape and surface properties. The thermal load in precision cutting is low and highly dynamic, making it challenging to measure. Commercially available thermocouples usually have a time constant that is higher than the time between single cuts. High-resolution thermographic imaging is not fast enough to measure the dynamic of the temperature propagation as well. Thus, an approach with directly deposited thermocouples on the surface of the workpiece with minimum time constant due to thin film electrical insulation was realized. Previous experiments have shown the suitability of this approach for high-speed, high-resolution temperature measurement [11]. In those experiments, the thermocouple was formed from a screen-printed silver electrode and the workpiece. However, the sensitivity of the thermocouple was uncertain, and the use of the workpiece as a second electrode led to severe noise during the crucial contact of the machining tool and the workpiece. Thus, in this work, we choose to deposit a fully insulated two-electrode thermocouple from pure metals.

## 2. Materials and Methods

### 2.1. Methods for Temperature Measurement

Temperature measurement can either be implemented by resistive or thermo-electric sensing [12]. Resistive temperature measurement is based on the dependency of electrical resistivity on temperature. Common resistive temperature sensors, such as Pt100 or Ni1000, are commercially available and well established. However, resistive sensors will always be sensitive to deformation along with temperature. As we expect temperature as well as strain to occur simultaneously in machining processes near the machined surface, resistive temperature measurement is not applicable [13]. Thermo-electric temperature measurement by thermocouples is more complex than resistive measurement, yet it is non-sensitive to strain. Thus, we decided to fabricate thermocouples on the surface of workpieces for temperature measurement. The direct deposition on the surface of the workpiece allows a minimal thermal decoupling between the sensor and the workpiece caused by the electrical insulation only.

The thermo-electric voltage of a thermocouple is given by

(1) $$U_{th}=\left|S_{1}-S_{2}\right|\cdot \Delta T$$

with *S*_1_ and *S*_2_ being the Seebeck coefficients of the materials used for fabrication of the electrodes and ΔT being the difference in temperature from the joint of the electrodes to the contact area.

### 2.2. Sensor Design 

#### 2.2.1. Workpiece and Sensor Dimensions

The workpieces for precision machining have a size of 15 mm × 33 mm × 2 mm (width × height × thickness). We investigated workpieces made of steel (42CrMo4), aluminum (EN AW-5083), and copper (Cu-OF). The workpieces have been cut from rolled sheet material.

**Figure 1:**
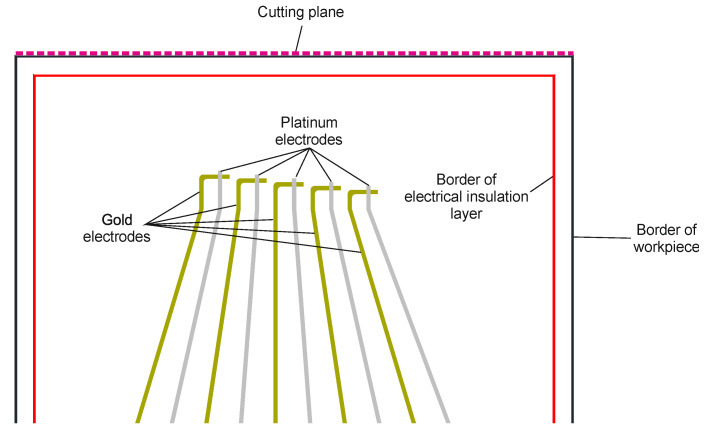
Top view of thermocouples on the workpiece. Five thermocouples are formed from gold and platinum electrodes with varying distance to the cutting plane.

Five thermocouples are deposited on the surface of each workpiece, as shown in Figure 1. The electrodes are 500 µm wide with 500 µm spacing at the bottom part of the workpiece to allow easy electrical contact with a suitable connector. The contact area is not shown in Figure 1. The electrodes narrow down to a width of 100 µm in the measurement area. The two electrodes of the thermocouple have an overlapping measurement area of 100 × 100 µm². Each consecutive thermocouple on the workpiece is 100 µm further away from the cutting plane to allow for reconstruction of the temperature propagation in the workpiece. The Seebeck coefficients of gold and platinum are 6.5 and 0 µV/K, respectively.

#### 2.2.2. Sensor Fabrication

Microfabrication on small sheets of metal is challenging since most equipment is built for the use of standard silicon wafer substrates. The rolled material neither has a high-quality polished surface nor is the material compatible to many standard microfabrication technologies. Hence, standard processing involving lithography and etching cannot be used for fabrication.

**Figure 2:**
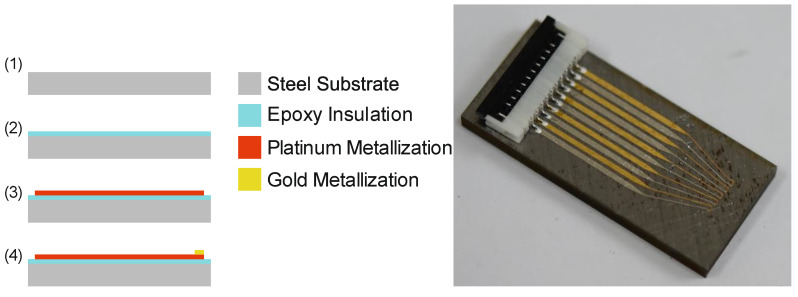
Fabrication process (**left**) and final workpiece (**right**).

The fabrication of the sensors can be divided into two major steps: (1) formation of an insulation layer and (2) the deposition of thermocouple electrodes. The fabrication is shown in Figure 2 (left). The deposition of ceramic dielectric layers such as SiO_2_, Al_2_O_3_, or ZrO_2_ has not been taken into consideration, since the available technical equipment was not able to fabricate pinhole-free, fully insulating layers of any of those materials on low-quality surfaces. Instead, the insulation layer was made of epoxy resin. Deposition was done by manual screen-printing of three layers of the epoxy (UHU Endfest 90 min). The thickness of the final insulation layer is approximately 3.5 µm.

Gold and platinum electrodes are sputter-deposited by a DC sputtering process with a power of 100 W for a time of 192 s and 200 W for a time of 204 s, respectively. The resulting layer thickness for both materials is approximately 300 nm. A shadow mask, which is made of a DRIE etched, 380 µm thick, double-side polished standard silicon wafer, is used to structure the electrodes during sputter-deposition. A resolution of 100 µm can easily be achieved by the use of shadow masks. A custom designed holder allows the contemporaneous coating of 11 workpieces on the area of a 150 mm wafer. Sufficient alignment between the shadow mask and the substrate is secured by corresponding tolerances of the holder, shadow masks, and substrates.

Electrical contacting is done by a Würth ZIF connector, which is glued on the surface of the substrate. The pins are connected to the electrodes with Panacol Elecolit 414 electrical conductive glue. The final workpiece is shown in Figure 2 (right).

### 2.3. Electronics Setup

**Figure 3:**
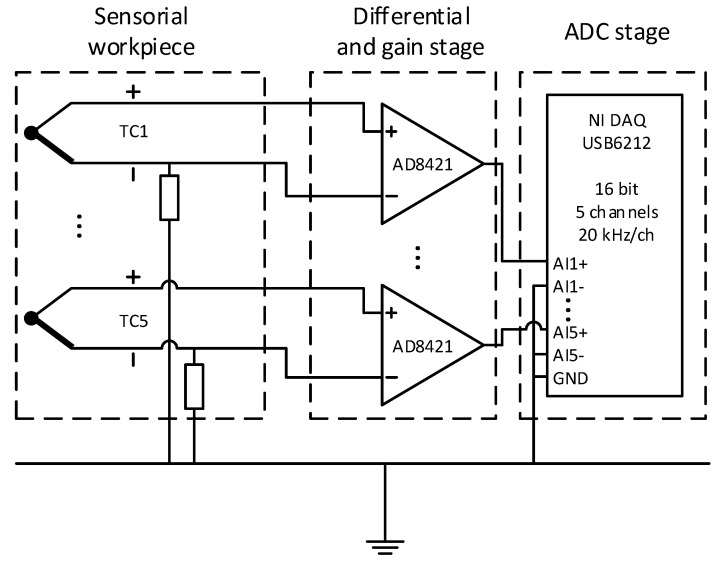
Electronics setup consisting of thermocouples, differential and gain stage, and analog-to-digital conversion stage.

A custom-built amplification circuit was designed to perform measurements. The circuit consists of 5 AD8421 high-performance instrumentational amplifiers. The signal was amplified by a factor of approximately 2010. The electrical connection to the thermocouples is established by Würth ZIF 10-pin connectors and matching flat cables. The analog-to-digital signal conversion is done by a National Instruments DAQ USB-6212 data acquisition unit in the differential input mode. All negative inputs are connected to the ground, while the positive inputs are connected to the output of the amplifiers. Figure 3 shows the electronics setup.

### 2.4. Setup for Machining

The machining experiments were performed on a Moore Nanotech 350FG (Swanzey, USA). This ultra-precision machine tool has an air-bearing main spindle and a hydrostatical supported Y-axis. In order to avoid environmental errors, the machine was operated under laboratory conditions at a controlled temperature of 20 °C. The machine is placed on a separate foundation with additional active damper units. Thus, temperature and vibration influences from outside are almost excluded. To favor a machining geometry for a relatively simple simulation of the process, the experiments were carried out as free quasi-orthogonal cutting. The uncoated submicron carbide grade tool was mounted on the main spindle to rotate at a swing diameter of 138 mm. A new tool of the type Arno 31602 AK10 (Ostfildern, Germany) was used on each workpiece. The workpieces were secured in a workpiece holder, which offered a guidance for the required wired connection between the temperature sensors and the measuring equipment with reduced interference caused by other machine components. To measure the process forces, the workpiece holder was mounted on a Kistler MiniDyn 9119AA1 (Sindelfingen, Germany). This three-component dynamometer offers a precise method to measure the process forces in three orthogonal directions with a sampling rate of 10 kHz.

**Figure 4:**
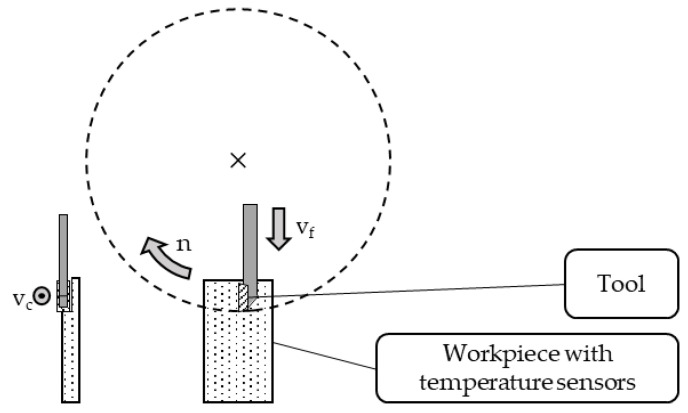
Schematic representation of the fly-cutting process. To validate the measurements of the newly developed sensors, a variation of the machining process was performed to ensure varying temperatures. The workpiece material was altered between steel (42CrMo4), aluminum (EN AW-5083), and copper (Cu-OF). Another variation in resulting temperatures was realized variating the process parameters cutting speed and feed according to Table 1.

During the machining experiments, for each workpiece, a new tool with a straight cutting edge and a cutting edge radius of r_β_ = 16 μm was applied. In the machining process, the rotating tool was plunged downwards into the workpiece with a cutting width of 1 mm and the respecting feed velocity v_f_ to achieve a feed f of 4.5 µm to 18 µm per revolution of the tool. A schematic representation of the machining process is shown in Figure 4. A photography of the machining setup and a video of the machining process are available as Supplementary Data S1 and S2, respectively.

Each combination of process parameters was performed three times as machining experiment, with copper being the exception, where due to the number of workpieces, only two experiments per parameter combination were conducted. Each workpiece had five temperature sensors in different depths related to the feed direction. This experimental implementation results in a database of 10 to 15 measured time-dependent temperatures for one parameter combination, which accommodates for statistical replication. An increase in the measured temperature was expected subsequently with each tool engagement in each experiment and generally with the increase of cutting speed or feed. The feed stopped at a depth at which the measurement areas of the temperature sensors were definitely reached and subsequently destroyed in the machining process. This ensures a measurement as near as possible to the machining zone, right before the sensor is destroyed with further tool movement in the feed direction.

### 2.5. Modeling and Simulation

The simulation is divided into two steps. In the first step, the cutting process itself is simulated and temperature over the workpiece surface and the corresponding heat flux is calculated as well. In the second step, the behavior of the heat flux from the workpiece to the dielectric epoxy layer and the sensor is simulated.

#### 2.5.1. Material Model

A thermo-visco-plastic material model was used in the simulations. The material behavior included isotropic hardening according to the well-known Johnson–Cook model (JC). The thermo-physical and mechanical properties of all materials were taken from the literature [14,15] and are listed in Table 2 and Table 3. The resulting yield stress in the JC model is given by

(2) $$\bar{\sigma }=\left[A+B\varepsilon_{p}^{n}\right]\left[1+Cln\left(\frac{\dot{\varepsilon }}{{\dot{\varepsilon }}_{0}}\right)\right]\left[1-{\left(\frac{T-T_{0}}{T_{m}-T_{0}}\right)}^{m}\right].$$

The material parameters are *A*, *B*, *C*, *m*, and *n* with ${\dot{\varepsilon }}_{0}$ as the reference strain rate, and $T_{m}$ and $T_{0}$ as the melting and room temperature, respectively.

#### 2.5.2. Friction Model

There is no constant coefficient of friction for the interaction between the cutting tool and the workpiece as the pressure at the contact surface is very high. Based on the friction model by Zorev [16], the friction properties at the tool-to-chip interface were defined as:

(3) $$\tau =\min(\tau_{Y},\mu \bar{\sigma })$$

(4) $$\tau_{Y}=\bar{\sigma }/\sqrt{3}$$

The shear stress τ is limited either by the shear flow stress $\tau_{Y}$ of the material or by Columb friction behavior with a friction coefficient of *μ* = 0.6 [17]. Table 4 lists the friction model parameters.

#### 2.5.3. Boundary Conditions and Mesh

**Figure 5:**
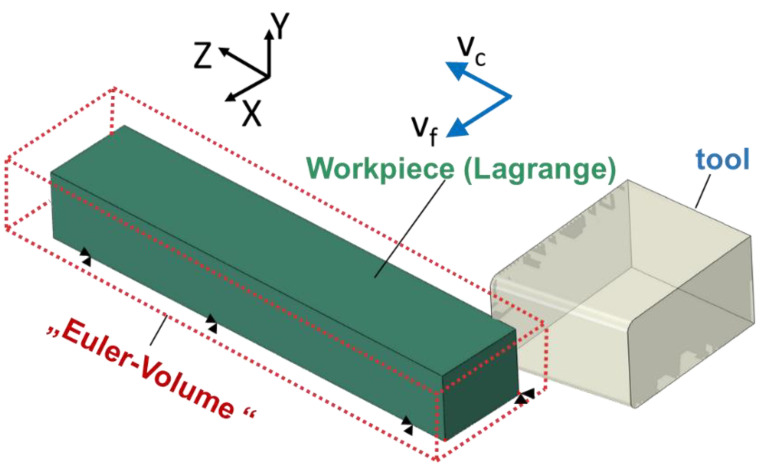
Schematic representation of cutting tool and workpiece.

The workpiece is fixed in space while the tool moves in Euler space during cutting. The cutting tool is connected to a reference point on the axis of rotation of the tool. All boundary conditions, such as rotation, are applied to the reference point. To reduce calculation time, the dimensions of the workpiece used in the simulations were reduced to 2 mm × 1.5 mm × 10 mm (X × Y × Z), and only the first five cuts have been considered in this work. Figure 5 shows the orientation of the workpiece and tool in space.

Temperature displacement solid continuum elements were used for modeling the thermal and mechanical behavior of the workpiece. The tool was meshed with C3D4T-Lagrange tetrahedral elements. Euler volume meshing was realized with EC3D8RT elements with reduced integration and hourglass control. Based on the recommendations given in [18] and on our own preliminary investigations, an element size of 18 µm was chosen for the Euler volume. The initial temperature of workpiece and tool was set to 20 °C (room temperature). Movements of the workpiece in all directions (displacement along the X-, Y-, Z-axis and rotation about the X-, Y-, Z-axis) were fixed.

The tool was fixed in the Z- and Y-direction. In the simulations, a feed of f_1_ = 18 µm and f_2_ = 36 µm, and a spindle speed of n = 1058.73 min^−1^ (i.e., as cutting velocity of v_c_ = 459 m/min) were chosen.

#### 2.5.4. Mass Scaling of Workpiece for Computational Efficiency

The 3D Eulerian FE analysis is computationally intensive and time consuming. The mass scaling technique is often used to reduce computation time. In addition to mass scaling, time scaling can be used to further reduce computation time in coupled Eulerian–Lagrangian modeling [19].

In mass as well as in time scaling, the thermal time constant has to be maintained by adjusting the thermal properties of the material accordingly. The mass is scaled by replacing the density *ρ* with the fictitious density ρ* (*k_m_* > 1).

(5) $$\rho^{*}=k_{m}$$

Replacing the density *ρ* by fictious density ρ* results in a change of the thermal time constant. Thus, the fictious specific heat c_p_* must be used to counter this effect.

(6) $$c_{p}^{*}=c_{p}k_{m}^{-1}$$

A mass scaling factor of *k_m_* = 25 results in good agreement with experimental values of mechanical forces. Increasing the scaling factor leads to an increase in deviation from experimental results. The computation time at a scaling factor of 25 was approximately 18 h. The optimal scaling factor in this work is similar to the factor for milling simulation, which was determined in [20].

#### 2.5.5. Simulation of Sensor Response

**Figure 6:**
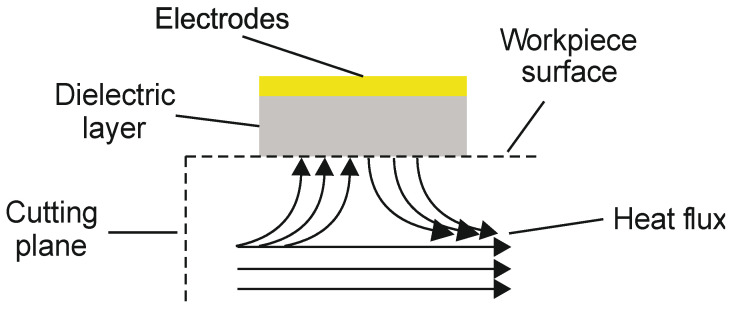
Setup of the simulation of the sensor signal from the heat flux in the workpiece.

From the simulation of the cutting process, the temperature on the surface of the workpiece as well as the corresponding heat flux could be calculated. The major challenge in the simulation of the sensor signal was the calculation of the thermal insulation of the thermocouple electrodes due to the dielectric layer. The layer was approximately 3.5 µm thick, which was measured by tactile step measurement. In order to calculate the signal of the sensor, a 2D model of the cross-section at the intersection of the thermocouple electrodes was used. The model is shown in Figure 6. At the bottom of the dielectric layer, the heat flux from the cutting simulation was applied. While the tool was in contact with the workpiece, heat was generated in the contact zone. A temperature wave from the contact zone to the bottom part of the workpiece was induced. While the tool was not in contact with the workpiece, which was the case the majority of the time, the heat dissipated in the workpiece. Thus, during heat generation, there was a heat flux from the workpiece to the thermocouple and, once the heat rapidly dissipated into the workpiece after contact, there was a heat flux from the thermocouple to the workpiece.

The crucial parameter for the simulation of the sensor response is the thermal conductivity of the materials. The thermal conductivity of each material can be found in Table 5.

## 3. Results

### 3.1. Measurement Results

**Figure 7:**
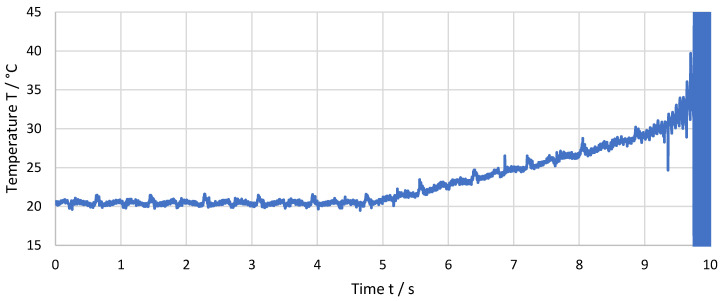
Measured temperature over the last 10 s before the destruction of the thermocouple for a feed of f = 36 µm.

Temperature measurement was affected by high noise levels from the machining environment. Thus, all results have were digitally filtered using a 3rd order Bessel low-pass filter with a cut-off frequency of 100 Hz, which does not falsify the temperature signal. Low-frequency noise is still visible. Figure 7 shows the measured and filtered temperature within the last 10 s before the destruction of the thermocouple for a feed f = 36 µm and a cutting speed of v_c_ = 459 m/min. The temperature starts to rise slowly when the cutting plane has reached a certain distance to the thermocouple. Shortly before the thermocouple is destroyed, single peaks in temperature become visible.

**Figure 8:**
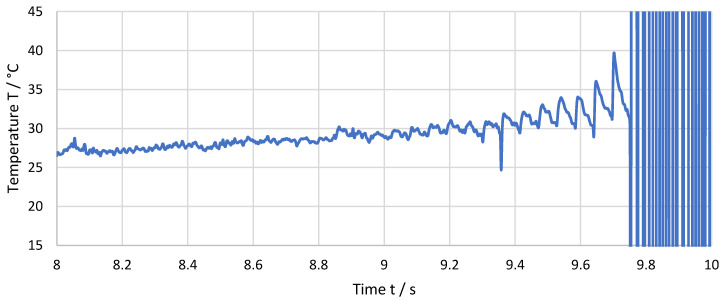
Measured temperature over the last 2 seconds before the destruction of the thermocouple for a feed of f = 36 µm.

Figure 8 shows an enlarged view of the last 2 seconds before the destruction of the thermocouple. The cutting speed of v_c_ = 459 m/min is achieved at an approximately 1000 rpm rotational speed of the spindle. This translates to a cutting frequency of approximately 17 Hz. The temperature peaks are clearly visible for the last 10 cuts. The frequency of the peaks corresponds to the cutting frequency and, therefore, the individual peaks can be related to single cuts. For each cut, the temperature rises fast to a temperature maximum and then drops back at a lower change rate almost to the temperature before the cut.

The non-computed measurement data as recorded during the experiment as well as spectral analysis data of Figure 7 and Figure 8 are available as Supplementary Material S3 and S4, respectively.

**Figure 9:**
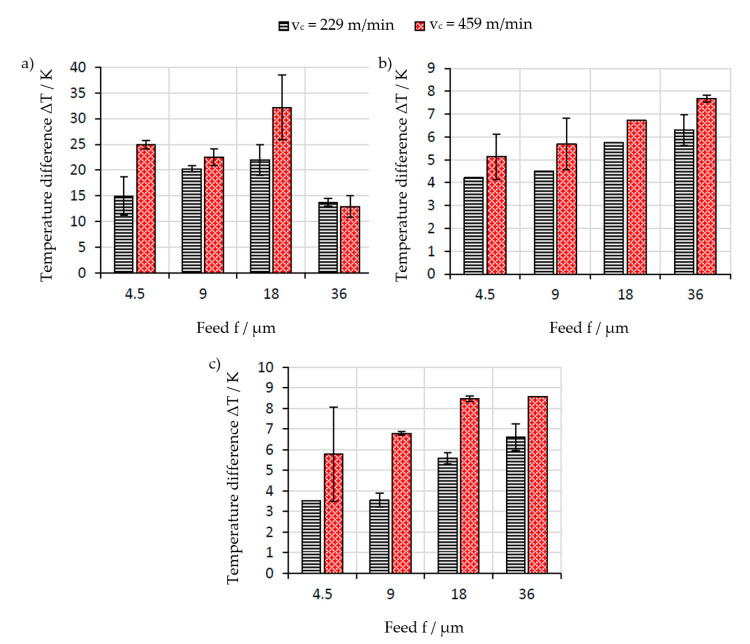
Average maximum temperature difference from room temperature for (**a**) steel, (**b**) aluminum, and (**c**) Cu-OF workpieces at different feeds and cutting speeds.

The average maximum temperature difference for different materials, feeds, and cutting speeds (here expressed as rotational speeds n) are summarized in Figure 9. The maximum temperature difference is mostly increasing with cutting speed and feed. The standard deviation is low, indicating a good repeatability of the experiments and high reliability of the data. However, against the expectations, the temperature decreases noticeably for a feed of f = 36 µm when cutting the steel workpieces. The reason for the decrease in temperature is yet to determined.

### 3.2. Simulation Results

Process simulations have been carried out for steel workpieces at a cutting speed v_c_ = 459 m/min and feeds f_1_ = 18 µm and f_2_ = 36 µm. Those parameters yielded the most interesting results in temperature measurements showing the highest average temperature and an unexpected drop in maximum temperature, respectively. The last five cuts before the destruction of the thermocouple were simulated.

**Figure 10:**
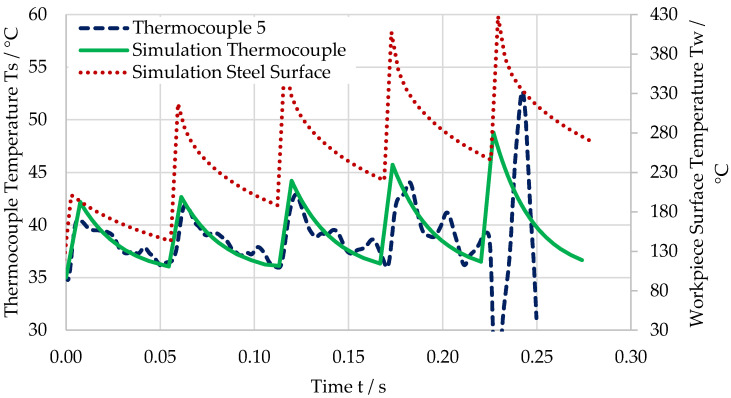
Temperature for the last five cuts before the destruction of the thermocouple for a feed of f = 18 µm. Measured temperature (dashed blue line), simulated temperature at workpiece surface (dotted red line), and calculated temperature of thermocouple (solid green line).

Figure 10 shows the temperature of one of the thermocouples (dashed blue line), the simulated temperature at the workpiece surface below the thermocouple (dotted red line), and the calculated temperature of the thermocouple (solid green line) for a feed of f = 18 µm. Measured and simulated temperatures show a very good match qualitatively and quantitatively. It is noticeable that the temperature at the workpiece surface is approximately 10 times higher than the temperature measured and simulated at the sensor. This is likely resulting from the poor thermal conductivity of the dielectric layer.

**Figure 11:**
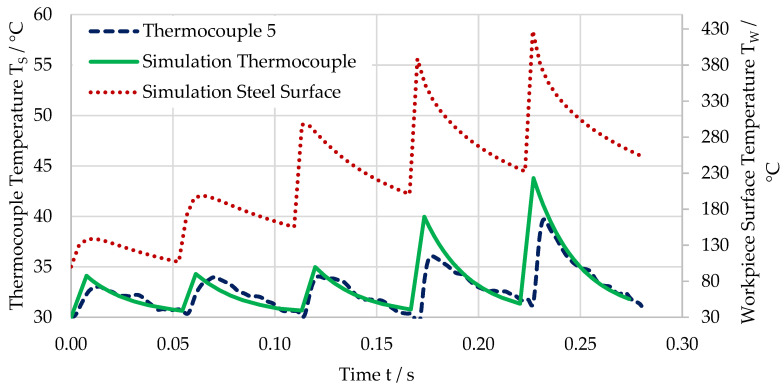
Temperature for the last five cuts before the destruction of the thermocouple for a feed of f = 36 µm. Measured temperature (dashed blue line), simulated temperature at workpiece surface (dotted red line), and calculated temperature of thermocouple (solid green line).

Figure 11 shows the temperature of one of the thermocouples (dashed blue line), the simulated temperature at the workpiece surface below the thermocoule (dotted red line), and the calculated temperature of the thermocouple (solid green line) for a feed of f = 36 µm. Again, the measured and simulated temperatures show a very good match, and the workpiece temperature is approximately 10 times higher than the measured temperature. Even the less distinct peak in temperature after the first three cuts can be seen in the measurement data.

## 4. Discussion

The temperature measurement with thermocouples, which consists of pure metal electrodes, is very reliable. The impact of every single cut during machining can be seen once the tool is close to the thermocouple. The spatial as well as the temporal resolution of the resulting temperature measurements are high enough to accommodate the investigated highly dynamic process. However, a smaller time constant of the thermocouples would be appreciated. The thermal insulation of the thermocouple from the workpiece, which is caused by the dielectric insulation layer, is an issue. It might be an option in future experiments to form the insulating layer from a material that has higher thermal conductivity.

When compared to results from prior experiments in which a screen-printed silver electrode and the workpiece formed the thermocouple, the results match fairly well. However, the use of the workpiece as the second electrode of the thermocouple was meant to eliminate the influence of a dielectric layer on the measured temperature. Yet, the temperatures measured in both thermocouple setups are very similar. Since the use of a pure metal thermocouple should result in a high reliability of the data, we believe that either the use of screen-printed electrodes or the workpiece as an electrode is not suitable for temperature measurement.

The simulated temperature at the surface of the workpiece is approximately 10 times larger than the temperature, which is measured by the thermocouples. Taking into consideration the thermal insulation due to the dielectric layer, the measurement and simulation results match very well. The division of the simulation into two steps, namely cutting process simulation and actual sensor signal simulation, might be a source for errors. However, each single simulation step itself is based on well-known and established simulation methods. In future work, the simulation steps will be merged into one simulation that can calculate both cutting process and sensor behavior at once.

## 5. Conclusions

The results from this work are very valuable in the overall scope of the SFB TRR, which includes the determination of local material loads in machining processes and their correlation with material modifications. As of now, finite element simulation is the only way to determine local material loads, since these local loads are not measurable directly in situ during a machining process. Yet, the simulation has to be validated by experimental data whenever possible. The temperatures, which have been measured in this work, validate our process model. Our results show that the currently used simulation methods provide results that match the temperatures and therefore a material load in quantity and quality. Thus, it can be stated that local material loads, which are determined by simulation, match the loads that will occur in the cutting process. In further research within the SFB TRR, the material model can be used to identify local material loads. The correlation of those local loads with material modifications offer a great potential to solve the inverse problem of manufacturing.

## Figures and Tables

**Table 1:** Variation of workpiece materials and process parameters.

Workpiece Material	Steel(42CrMo4)		Aluminum(EN AW-5083)		Copper(Cu-OF)	
Cutting Speed v_c_	229 m/min			459 m/min		
Feed f	4.5 µm	9 µm		18 µm		36 µm

**Table 2:** Johnson–Cook model coefficient for AISI 4140 [14,15].

JC Constitutive Model	A (MPa)	900
	B (MPa)	650
	C	0.034
	m	0.328
	n	0.45
	${\dot{\varepsilon }}_{0}$ (s^−1^)	0.001
	$T_{m}$ (°C)	1536
	$T_{0}$ (°C)	20

**Table 3:** Physical and mechanical properties of workpiece and tool [14,15].

E (GPa)	AISI 4140	204
ν	AISI 4140	0.3
ρ (kg/m³)	AISI 4140	7850
	tungsten carbide	15,000
k (W/mK)	AISI 4140	47
	tungsten carbide	46
λ (1/K)	AISI 4140	12.3∙10^−6^
	tungsten carbide	4.7∙10^−6^
C_p_ (J/kgK)	AISI 4140	475
	tungsten carbide	203
Inelastic heat fraction		0.9

**Table 4:** Friction model parameters.

Columb Friction Coefficient	0.6
Friction energy converted to heat (%)	100
Fraction of converted heat distributed to tool surface	0.5

**Table 5:** Thermal conductivity of 42CrMo4 steel, epoxy insulation layer, and thermocouple electrodes.

42CrMo4 Steel	λ_T,Steel_ (W/m*K)	44
Uhu Endfest expoxy resin	λ_T,Steel_ (W/m*K)	0.24
Gold	λ_T,Steel_ (W/m*K)	314

## Data Availability

The data presented in this study is partially available in supplementary materials. Additional data presented in this study is available on request from the corresponding author.

## Supplementary Materials

The following are available online at https://www.mdpi.com/1424-8220/21/4/1530/s1, Figure S1: Photograph of measurement setup in cutting machine, Video S2: Video of cutting process, Measurement Data S3: Measurement data of Figure 7 and Figure 8 as recorded, Figure S4: Frequency spectra of Figure 7 and Figure 8.
