# Supplementary figures and images for: An Investigation on High-Resolution Temperature Measurement in Precision Fly-Cutting
Source: Sensors (Basel). 2021 Feb 23;21(4):1530. doi: 10.3390/s21041530 (PMC7926914; doi:10.3390/s21041530)

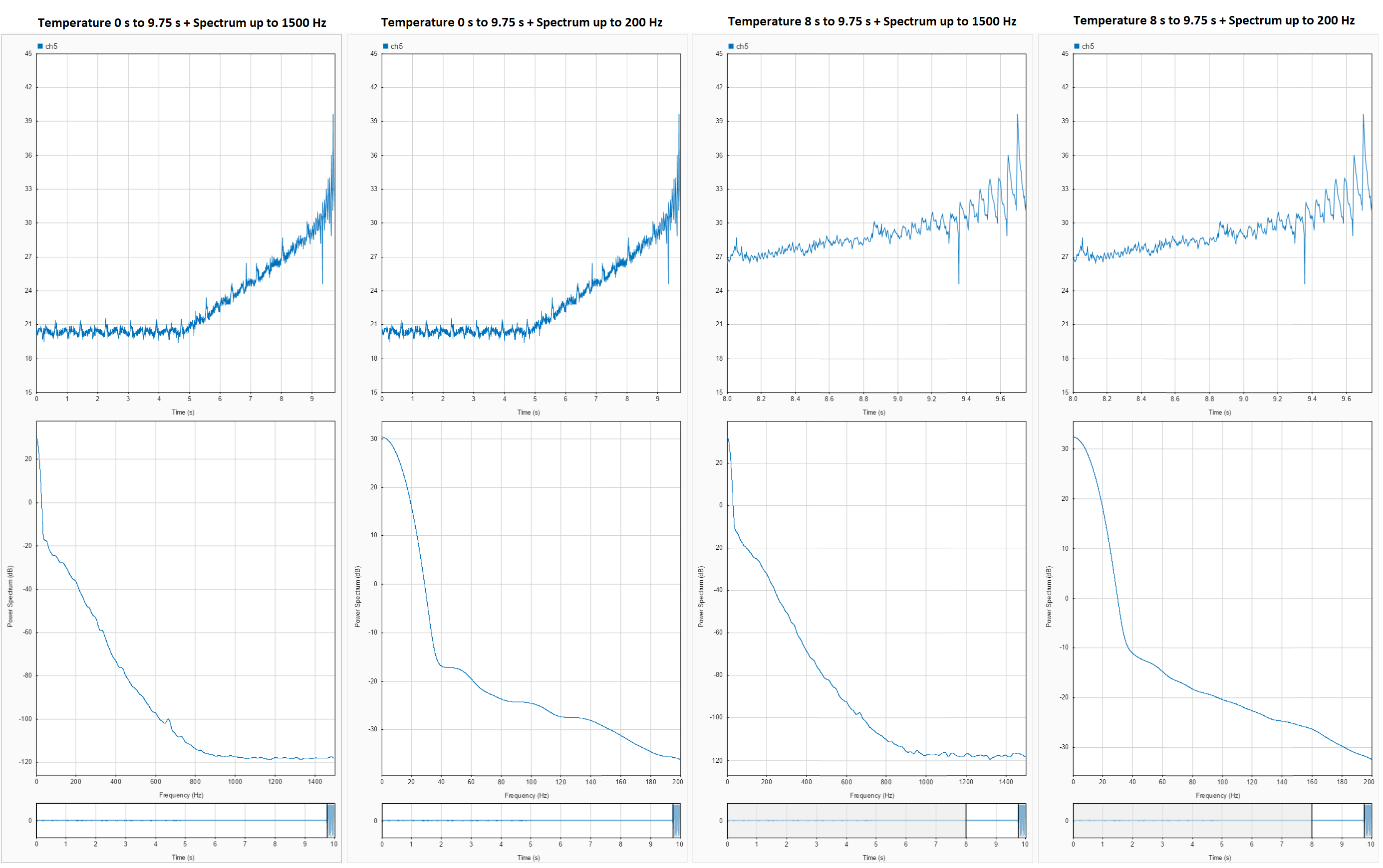

Supplement: Supplementary file 1 [file sensors-21-01530-s001.zip › S4_Frequency spectra of figure 7+8.PNG]
